# Burnout syndrome among health care professionals in the context of the COVID-19 pandemic

**DOI:** 10.47626/1679-4435-2023-1134

**Published:** 2024-09-24

**Authors:** Letícia Menezes de Oliveira, Selene Cordeiro Vasconcelos, Fernando Lannes Fernandes, Ana Lúcia Belarmino de Araújo, Gesualdo Gonçalves de Abrantes, Ana Karina Bezerra Pinheiro

**Affiliations:** 1 Enfermagem, Universidade Federal da Paraíba (UFPB), João Pessoa, PB, Brazil; 2 Programa de Pós-Graduação em Enfermagem (UFPB), João Pessoa, PB, Brazil; 3 Geografia, University of Dundee, Dundee, Scotland; 4 Enfermagem, Universidade Federal do Ceará (UFC), Fortaleza, CE, Brazil

**Keywords:** Professional burnout, health personnel, COVID-19, esgotamento profissional, pessoal de saúde, covid-19

## Abstract

**Introduction:**

Burnout syndrome, a mental illness caused by stressful work conditions, is prevalent among health professionals. In addition to existing risk factors, the COVID-19 pandemic introduced new ones, such as fear of infection and concern about the availability of personal protective equipment.

**Objectives:**

This study’s purpose was to verify the prevalence and correlates of burnout syndrome among health professionals in the context of the pandemic.

**Methods:**

This quantitative study was conducted between April and June 2021 using the Google Forms platform. A semi-structured questionnaire and the *Questionário para la Evaluación del Syndrome de Quemarse por el Trabajo* were applied.

**Results:**

A total of 93 health professionals participated. Those who fought on the frontline against COVID-19 were younger and had less professional experience, longer work hours, daily contact with a greater number of patients, lower scores in the disillusionment dimension, and higher scores in the emotional exhaustion dimension. Significant correlations were found between age and disillusionment, emotional exhaustion, and indolence. Less professional experience was also correlated with psychological distress.

**Conclusions:**

Age and length of experience were significantly associated with burnout, given that younger and less experienced professionals generally worked on the frontline against COVID-19.

## Abstract (pt)

**Introdução:**

A síndrome de *burnout* é uma doença psíquica ocasionada por situações desgastantes de trabalho, sendo prevalente entre os profissionais de saúde. Além dos fatores de risco já existentes para o seu desenvolvimento, a pandemia de covid-19 apresentou novos fatores de risco, como o medo de adquirir a doença e a preocupação com a disponibilidade de equipamentos de proteção individual.

**Objetivos:**

Verificar a prevalência da síndrome de *burnout* em profissionais de saúde no contexto da covid-19 e suas correlações.

**Métodos:**

Trata-se de um estudo quantitativo, realizado em 2021 por meio da plataforma Google Forms. Foram utilizados um questionário semiestruturado e o Cuestionario para la Evaluación del Síndrome de Quemarse por el Trabajo.

**Resultados:**

Participaram da pesquisa 93 profissionais de saúde. Os profissionais que participaram do enfrentamento à covid-19 possuíam menor idade, menor tempo de atuação profissional, maior carga horária semanal de trabalho, contato com maior número de pacientes diariamente, menor pontuação na dimensão ilusão pelo trabalho e maior pontuação na dimensão desgaste psíquico. Correlações significantes foram encontradas entre idade e ilusão pelo trabalho, desgaste psíquico e indolência. Além disso, menor tempo de atuação profissional foi correlacionado com desgaste psíquico.

**Conclusões:**

A idade e o tempo de atuação foram as variáveis que apresentaram significância nos testes estatísticos realizados, em que profissionais mais jovens e com menor tempo de atuação estavam em maioria no enfrentamento à covid-19, além de estarem associados ao *burnout,* corroborando para que fossem os profissionais mais susceptíveis a desenvolver a síndrome de *burnout.*

## INTRODUCTION

Work is fundamental for human evolution, since through it man develops his potential and modifies his environment according to the demands of each historical period, transforming raw materials into usable products.^1^ However, when focused solely on end products, work loses its fundamental essence, because rather than fulfilling personal needs it becomes a means to satisfy the goals of institutions. This contributes to occupational illness, both physical and mental.

Burnout, or professional exhaustion, syndrome is a form of mental illness caused by extreme work-related stress. It is an emotional disorder resulting from exhausting work situations and/or overwork, causing symptoms of mental and physical stress.^2^ Psychologist Herbert Freudenberg was among the first to describe burnout syndrome as a psychological disorder, characterizing it as a work-related reduction in energy, motivation, and effort.^3^

In Brazil, studies have shown a considerable prevalence of burnout syndrome among health care professionals. In a sample of 146 professionals from perioperative units, Munhoz et al.^4^ found a 10.3% prevalence of burnout syndrome, while Pereira et al.^5^ found a 13.2% prevalence among 282 emergency services workers.

Burnout syndrome can be the result of risk factors, such as work overload, excessive noise, and conflicts between team members, patients, and family members.^6^ In addition to these factors, during the COVID-19 pandemic, the fear of becoming infected and infecting others and concern about the lack of effective personal protective equipment were identified as mental health risk factors among health care professionals, whose physical condition and social relationships were greatly affected.^7^ Thus, in addition to causing new cases of burnout, the pandemic was an aggravating factor for health care professionals already suffering from the syndrome.

Early detection and monitoring of burnout syndrome among health care workers is becoming even more necessary. Strategies to promote mental health and prevent mental illness in health care institutions require occupational health professionals, such as nurses, and timely referral to effective treatment. Thus, this study aims to help bring about changes in work practice, since professionals often neglect their own care for that of patients. The research question was: “What is the prevalence of burnout syndrome among health care professionals in the context of COVID-19 and its associated variables?”

## METHODS

This analytical, quantitative cross-sectional study complied with Strengthening the Reporting of Observational Studies in Epidemiology methodological guidelines.^8^ We used the Google Forms platform, which allows participants to fill out an instrument and receive a response from researchers. The study was publicized on social media but, due to the difficulty of recruiting participants, the snowball strategy was used, ie, the link was sent via WhatsApp to a health professional, who then forwarded it to colleagues, and so on.

Data were collected between April and June 2021, during the critical phase of the pandemic, a period of intense uncertainty about the disease and changes in patient management and the work process due to transmission reduction measures and high mortality rates. The inclusion criteria were health professionals of either sex who worked in health services during the COVID-19 pandemic. When duplicate submissions were received, only the first was considered in the analysis.

A semi-structured questionnaire was used to obtain sociodemographic data and work-related personal information, and the *Cuestionario para la Evaluación del Síndrome de Quemarse por el Trabajo* (CESQT) was applied.^9^ The CESQT assesses burnout syndrome through 20 questions covering 4 dimensions: disillusionment, defined as a lack of motivation to achieve goals at work; emotional exhaustion, defined as work-related fatigue due to relationship problems; indolence, defined as indifference towards patients; and guilt, which is produced through attitudes towards the people one interacts with.^9^ Scores < 2 in the disillusionment dimension and scores ≥ 2 in the other dimensions indicate burnout syndrome. Professionals with burnout syndrome are subdivided into 2 profiles: (1) those scoring <2in the guilt dimension, and (2) those scoring ≥ 2 in the guilt dimension, indicating inability to work.^9^

The data were analyzed in IBM SPSS Statistics 20.0, with descriptive and inferential statistics through the Spearman correlation test and the Mann-Whitney U test; p < 0.05 was considered statistically significant. The study was approved by the institutional human research ethics committee (decision 31659220.3.0000.5188) and complied with National Health Council Resolution 466/2012,^10^ as well as Federal Nursing Council Resolution 564/2017 (the Code of Ethics for Nursing Professionals).^11^ The initial segment of the instrument was the informed consent form, which explained the study objectives, the risks and benefits of the study, and guaranteed participant anonymity.

## RESULTS

The sample consisted of 93 health professionals from the northeastern and midwestern regions of Brazil who completed the instrument. Their age ranged from 23 to 68 years (mean, 41 years) and they had between 1 and 45 years of professional experience. Their workload ranged from 20 to 88 hours per week, during which they had contact with up to 300 patients (median daily patients, 20).

The majority of the participants were women (n = 76; 81.7%), were married (n = 46; 49.5%), were trained as nurses (n = 52; 55.9%), worked in urgent or emergency care (n = 23; 24 .7%), had a graduate degree (n = 80; 86%), were on the frontline against COVID-19 (n = 59; 63.4%), and had no other job (n = 47; 50.5%).

The mean scores for each CESQT domain were: disillusionment, 3.1 points; emotional exhaustion, 2.3 points; indolence, 1.1 points; and guilt, 1.1 points. Most of the participants (70 [75.3%]) had burnout syndrome, of whom 60 (64.5%) were profile 1 and 10 (10.8%) were profile 2.

There were significant differences between those who did and did work on the frontline against COVID-19. Those who did were younger (p = 0.009), had less professional experience (p = 0.050), a greater weekly workload (p = 0.039), daily contact with a greater number of patients ( p = 0.046), lower disillusionment scores (p = 0.011), and higher emotional exhaustion scores (p = 0.014) (Table 1).

**Table 1:** Comparison between participants who were involved in combating COVID-19 or not, 2022

	Mann-Whitney U	p-value
**Age**	609.000	0.009*
Length of experience	684.000	0.050*
Workload	674.000	0.039*
Daily patients	681.000	0.046*
Disillusionment	615.500	0.011*
Emotional exhaustion	625.000	0.014*
Indolence	809.500	0.371
Guilt	762.000	0.193

* Statistically significant result.

The Spearman test showed significant correlations between the following variables: younger age and lower disillusionment (p = 0.042); greater emotional exhaustion (p = 0.002) and greater indolence (p = 0.037); and lower professional experience and higher emotional exhaustion (p = 0.045). The CESQT dimensions were intercorrelated, including a negative correlation with disillusionment: ie, as disillusionment increased, the others domains decreased and vice versa (Table 2).

**Table 2:** Spearman correlation results among between study variables, 2022

		Age	Length of experience	Workload	Daily patients	Disillusionment	Emotional exhaustion	Indolence	Guilt
Age	**Correlation**		0.841	−0.036	−0.057	0.211	−0.315	−0.217	−0.071
	**p-value**		0.000*	0.729	0.584	0.042*	0.002*	0.037*	0.500
Length of experience	**Correlation**	0.841		0.037	−0.163	0.122	−0.207	−0.188	−0.097
	**p-value**	0.000*		0.725	0.119	0.242	0.046*	0.071	0.357
Workload	**Correlation**	−0.036	0.037		0.067	0.057	-0.025	−0.071	−0.086
	**p-value**	0.729	0.725		0.524	0.586	0.814	0.500	0.410
Daily patients	**Correlation**	−0.057	−0.163	0.067		−0.081	0.148	−0.087	0.106
	**p-value**	0.584	0.119	0.524		0.440	0.158	0.409	0.314
Disillusionment	**Correlation**	0.211	0.122	0.057	−0.081		−0.457	−0.388	−0.175
	**p-value**	0.042*	0.242	0.586	0.440		0.000*	0.000*	0.094
Emotional exhaustion	**Correlation**	−0.315	−0.207	−0.025	0.148	−0.457		0.476	0.309
	**p-value**	0.002*	0.046*	0.814	0.158	0.000*		0.000*	0.003*
Indolence	**Correlation**	−0.217	−0.188	−0.071	−0.087	−0.388	0.476		0.418
	**p-value**	0.037*	0.071	0.500	0.409	0.000*	0.000*		0.000*
Guilt	**Correlation**	−0.071	−0.097	−0.086	0.106	−0.175	0.309	0.418	
	**p-value**	0.500	0.357	0.410	0.314	0.094	0.003*	0.000*	

* Statistically significant result.

Those who worked on the frontline against COVID-19 (younger participants and those with less professional experience) were more susceptible to burnout syndrome, being less disillusioned and experiencing greater emotional exhaustion and indolence.

## DISCUSSION

In the context of the COVID-19 pandemic, there was a higher prevalence of burnout syndrome among younger health care workers, given that they were the bulk of those on the frontline against COVID-19. Older age appeared to be a protective factor against work-related psychological distress, which may have been because older workers were allocated to other sectors due to risk reduction protocols. Therefore, younger professionals with less experience were more susceptible to burnout, suffering greater exhaustion and feelings of indifference.^9^ This shows the importance of occupational therapy strategies that investigate occupational stress and mental health status among health care workers.

A study conducted by Dutra et al.^12^ before the pandemic found that age was negatively correlated with emotional exhaustion and was positively correlated with personal fulfillment, indicating that younger professionals are more likely to become emotionally exhausted, while older professionals are more likely to find personal fulfillment. During the pandemic, Wang et al.^13^ determined the profile of professionals most affected by burnout syndrome. The characteristics included age < 29 years, < 5 years of professional experience, and those with lower education (eg, still finishing an undergraduate degree).

The CESQT results most associated with burnout syndrome among professionals who combated COVID-19 were high emotional exhaustion and low disillusionment. During the COVID-19 pandemic, stress, work overload, and low professional satisfaction were identified among health care workers.^14^ This resonates with our results, considering that low professional satisfaction was measured as lower scores in the disillusionment dimension.

Although working on the frontline against COVID-19 has been associated with work overload,^14^ In the present study, the workload variable was not significantly correlated with any CESQT dimension. Recent studies have confirmed that excessive work hours are a source of stress in the work environment, principally due to a reduction in the number of health care workers.^15^

It can be concluded that younger health care workers are significantly more liable to burnout syndrome, as are those with less professional experience, which is concerning since the occupational mental health of younger professionals is often neglected. This is important since they will replace older workers who are sustaining the country’s health care system. Hence, the number of disabled health workers may rise, further weakening the mental health of those who are already affected by burnout syndrome.

This study was limited by the difficulty of recruiting participants, obtaining only a small fraction of health care professionals.

## CONCLUSIONS

Burnout syndrome was highly prevalent among our sample of health care professionals during the COVID-19 pandemic. There was a significant association between working on the frontline against COVID-19 and CESQT dimension scores. CESQT scores were positively correlated with the number of daily patients and weekly workload, while they were negatively correlated with age and length of experience, ie, younger workers and those with less experience were more susceptible to burnout syndrome. This reflects work conditions during the COVID-19 pandemic, given that older workers were relocated to non-frontline sectors because their risk level was considered too high to work directly with patients.
